# Porous poly-l-lactide-co-ɛ-caprolactone scaffold: a novel biomaterial for vaginal tissue engineering

**DOI:** 10.1098/rsos.180811

**Published:** 2018-08-15

**Authors:** Reetta Sartoneva, Kirsi Kuismanen, Miia Juntunen, Sanna Karjalainen, Markus Hannula, Laura Kyllönen, Jari Hyttinen, Heini Huhtala, Kaarlo Paakinaho, Susanna Miettinen

**Affiliations:** 1 Adult Stem Cell Research Group, BioMediTech, Faculty of Medicine and Life Sciences, University of Tampere, Arvo Ylpönkatu 34, 4th Floor, 33520 Tampere, Finland; 2 Science Centre, Tampere University Hospital, Tampere, Finland; 3 Department of Obstetrics and Gynaecology, Tampere University Hospital, Tampere, Finland; 4 Faculty of Medicine and Life Sciences, University of Tampere, Tampere, Finland; 5 Faculty of Social Sciences, University of Tampere, Tampere, Finland; 6 Biomaterials and Tissue Engineering Group, BioMediTech, Faculty of Biomedical Sciences and Engineering, Tampere University of Technology, Tampere, Finland; 7 Computational Biophysics and Imaging Group, BioMediTech, Faculty of Biomedical Sciences and Engineering, Tampere University of Technology, Tampere, Finland

**Keywords:** poly-l-lactide-co-ɛ-caprolactone, neovagina, vaginal tissue engineering, vaginal epithelial cell, vaginal stromal cell, cell characterization

## Abstract

The surgical reconstruction of functional neovagina is challenging and susceptible to complications. Therefore, developing tissue engineering-based treatment methods for vaginal defects is important. Our aim was to develop and test a novel supercritical carbon dioxide foamed poly-l-lactide-co-ɛ-caprolactone (scPLCL) scaffold for vaginal reconstruction. The scaffolds were manufactured and characterized for porosity (65 ± 4%), pore size (350 ± 150 µm) and elastic modulus (2.8 ± 0.4 MPa). Vaginal epithelial (EC) and stromal cells (SC) were isolated, expanded and characterized with flow cytometry. Finally, cells were cultured with scPLCL scaffolds in separate and/or co-cultures. Their attachment, viability, proliferation and phenotype were analysed. Both cell types strongly expressed cell surface markers CD44, CD73 and CD166. Strong expression of CD326 was detected with ECs and CD90 and CD105 with SCs. Both ECs and SCs attached and maintained viability on scPLCL. Further, scPLCL supported the proliferation of especially ECs, which also maintained epithelial phenotype (cytokeratin expression) during 14-day assessment period. Interestingly, ECs expressed uroplakin (UP) Ia, UPIb and UPIII markers; further, UPIa and UPIII expression was significantly higher on ECs cultured on scPLCL than on cell culture plastic. In conclusion, the scPLCL is potential scaffold for vaginal tissue engineering and the results of this study further illustrate the excellent biocompatibility of PLCL.

## 1. Introduction

The vaginal defects can result due to numerous reasons. Failed embryonal fusion in the midline can cause genitourinary malformations such as vaginal agenesis [1]. Operative complications of pelvic organ prolapse surgery might lead to epithelial erosions or shortening of the vagina [2]; additionally, operative treatment of gynaecologic cancer may require removal of vaginal tissue [3,4]. In addition, transgender reconstructive surgery is a new and extremely challenging field of surgery requiring advanced reconstruction techniques [5,6]. Various surgical and non-surgical techniques have been used to reconstruct a functional neovagina. For vaginal agenesis, dilatation of vulvar tissue to create vagina is the first-line method. It is, however, a long process that takes patience and devotion, and yet is not always successful [7]. Different natural materials and acellular meshes such as amniotic membranes, peritoneal layers, recombinant artificial dermis and intestinal tissue have been studied for vaginal reconstruction [8–11]. Nevertheless, the non-vaginal tissues are not ideal for vaginal function and have problems such as shrinking, lack of lubrication, mucous secretion, stenosis formation and neovaginal prolapse depending on the graft tissue origin [8,12–16].

Recently, tissue engineering emerged as an alternative method for vaginal reconstruction. The selection of an appropriate biomaterial is essential; biomaterial should be biocompatible, flexible, suturable, easily moulded as a tubular structure, degrade without unfavourable tissue reactions and the mechanical properties of the scaffold should be close enough to the reconstructed vaginal tissue. Further, the scaffold for vaginal tissue engineering should adhere to the surrounding tissue, promote the viability and proliferation of both vaginal epithelial cells (EC) and stromal cells (SC), and the recovery of vaginal tissue architecture [17–19]. The poly-l-lactide-co-ɛ-caprolactone (PLCL) is known to be a biocompatible, elastic and flexible polymer, which is widely studied especially in soft tissue engineering applications, such as urothelial, vascular and neural tissue engineering [20–23]. The mechanical properties of the PLCL are more adequate to soft tissue engineering application when compared with other poly-α-esters such as polylactide and depend on the scaffold structure and manufacturing method [19,24,25]. Further, Vuornos *et al*. [25] have previously shown that the elastic modulus of supercritical carbon dioxide (scCO_2_) foamed PLCL (scPLCL) scaffold is 1.6 ± 0.6 MPa, which is closer to elastic modulus of vaginal tissue (6.65 ± 1.48 MPa) compared with, for instance, elastic modulus of braided PLA scaffold (280 ± 20 MPa). Even though the PLCL is widely studied in soft tissue engineering applications, at least best to our knowledge, the PLCL is not previously studied for vaginal tissue engineering. We wanted to study three-dimensional scaffold in order to facilitate the three-dimensional organization of cells [26]. For this study, we chose scCO_2_ foaming as a method for fabricating three-dimensional scaffold for vaginal tissue engineering in order to avoid any harmful solvents in fabrications process [27]. To our knowledge, the scCO_2_ foaming has not been previously studied for vaginal tissue engineering.

The aim of this study was to evaluate the suitability of scPLCL for vaginal tissue engineering by evaluating the morphology, viability and proliferation of vaginal ECs and SCs. Further, we also studied how the cells retain their viability and phenotype in co-cultures on scPLCL scaffold. Our hypothesis was that the scPLCL is a suitable scaffold structure for vaginal ECs and SCs and the cells maintain their viability and proliferate on the scPLCL.

## 2. Material and methods

### 2.1. Scaffold preparation

The polymer used in the porous scaffolds was 70/30 poly-l-lactide-co-ɛ-caprolactone (PLCL, 70 L/30CL; PURAC Biochem BV, Gorinchem, The Netherlands) with inherent viscosity of 1.6 dl g^−1^. The polymer was first melt-extruded into rods and in a second step foamed with scCO_2_ with a custom-fitted scCO_2_ reactor system (Waters Operating Corporation, Milford, MA, USA). The porous samples were cut into disc shape samples with a diameter of 5 mm and a height of 2–2.5 mm. The scaffolds were gamma irradiated for sterility with a minimum irradiation dose of 25 kGy.

### 2.2. Scaffold characterization with X-ray microtomography, differential scanning calorimetry and tensile testing

The scaffold structure was characterized with microtomography (µCT) imaging. The scaffolds (*n* = 3) were imaged with Xradia MicroXCT-400 (Zeiss, Pleasanton, CA, USA) X-ray device with 5.637 µm pixel size. Reconstruction was performed with the manufacturer's XMReconstructor software, and Avizo Software (Thermo Fisher Scientific, Waltham, MA, USA) was used for image processing and segmentation. Porosity and pore sizes were calculated with Fiji [28] using BoneJ plugin [29]. Interconnectivity of pores in PLCL scaffolds was calculated with developed Matlab (The MathWorks, Inc., Natick, MA, USA) script based on the pore size data.

The change in the crystallinity, measured as a change in melting enthalpy, was measured by a differential scanning calorimeter (DSC Q1000, TA Instruments, New Castle, DE, USA) for zero-week dry samples and samples incubated four weeks *in vitro* at 37°C in phosphate buffer solution (pH 7.6–7.9). Analyses were performed with 5–10 mg samples with the temperature range of 10–200°C with a heating rate of 20°C min^−1^.

The tensile tests were performed for scPLCL (*n* = 6) with thickness of 3.3 ± 0.3 mm, widths of 10.4 ± 0.4 mm and gauge lengths of 10 mm. The samples were strained with crosshead speed of 2 mm min^−1^ until 300% strain was reached. The elastic modulus of the samples was determined from the linear part of the resulting stress–strain curves. The mechanical tests were performed using Instron ElectroPuls E1000 (High Wycombe, UK) in ambient laboratory environment and in aqueous environment at 37°C with Instron's temperature-controlled fluid bath. Prior to testing in aqueous environment, the samples were incubated in phosphate buffer solution (pH 7.6–7.9) at 37°C for 48 h.

### 2.3. Cell isolation

For this study, human vaginal ECs and SCs were isolated from vaginal tissue pieces from three patients undergoing vaginectomy in Tampere University Hospital. The isolation protocol was modified from De Filippo *et al*. [30]. Briefly, the tissue sample was washed twice with Hanks' balanced salt solution (HBSS, Life Technologies, Thermo Fisher Scientific, Waltham, MA, USA) and cut into small pieces. The pieces were digested in solution containing 1.5 mg ml^−1^ of collagen cleaving collagenase type I (Life Technologies), and 4 mg ml^−1^ of dispase, separating the EC (Invitrogen, Thermo Fisher Scientific) for 60 min at 37°C water bath with shaker on. The digested tissue was filtered through the 100 m cell strainer (BD Biosciences, San Jose, CA, USA) and the resulting suspension was centrifuged. The digested tissue pieces and the resulting pellet were plated on separate CellBind T75 flasks (Sigma-Aldrich, St Louis, MO, USA) with EpiLife medium (Invitrogen) supplemented with 1% of EpiLife defined growth supplement (EDGS; Invitrogen), 0.1% of CaCl_2_ (Invitrogen) and 0.35% of antibiotics (100 U ml^−1^ penicillin and 0.1 mg ml^−1^ streptomycin (Lonza, BioWhittaker, Verviers, Belgium) and cultured at 37°C in a humidified atmosphere of 5% CO_2_ in air. After primary culturing, the cells were treated with TrypLE Select (Gibco, Thermo Fisher Scientific) for 2 min and the cells were passaged to T75 flasks (Nunc, Thermo Fisher Scientific) in DMEM/F12 (basic medium, BM; Thermo Fisher Scientific) supplemented with 5% human serum (Biowest, Nuaillé, France), 1% GlutaMAX (Life Technologies) and 1% of antibiotics (100 U ml^−1^ penicillin and 0.1 mg ml^−1^ streptomycin; Lonza), resulting human vaginal SC line. The remaining cells were treated a second time with TrypLE Select and passaged to T75 flasks with EpiLife medium, resulting human vaginal EC line. The cells were passaged when confluent and the vaginal ECs and SCs in passages 2–3 were used in all *in vitro* tests except flow cytometric analysis.

Prior to experiments, four different medium compositions; EpilLife, 3% HS in EpiLife, EpiLife and BM 1 : 1 and CnT prime CC (CELLnTEC Advanced Cell Systems AG, Bern, Switzerland) were tested with the ECs, SCs and vaginal-SC co-cultures for 7 days. According to the live/dead staining results (electronic supplementary material, figure S1), EpiLife was chosen as the culture medium for co-cultures.

### 2.4. Flow cytometric surface marker expression analysis

The human vaginal ECs and SCs (*n* = 3, passages 3–4) were harvested and analysed after cell culture with fluorescence-activated cell sorter (FACS; FACSAria Fusion Cell Sorter, BD Biosciences). Monoclonal antibodies against CD44-PE, CD73-PE, CD90-APC (BD Biosciences), CD105-PE (R&D Systems, Oxon, UK), CD133-PE (Miltenyi Biotech, Bergisch Gladbach, Germany), CD166-PE (BD Biosciences) and CD326-PE (Miltenyi Biotech) were used. In total, 10 000 cells per sample were analysed and unstained cell samples were used to compensate the background autofluorescence levels.

### 2.5. Cell seeding

Before the cell seeding, the scPLCL scaffolds were pre-incubated in medium at 37°C for 24 h in order to pre-wet the samples and placed on 48-well plates (NuncTM, Thermo Fisher Scientific). The cell culture studies were performed either by culturing vaginal ECs and SCs on separate scaffolds or co-culturing the ECs and SCs.

In separate culturing, 40 000 ECs or SCs in 10 μl of medium were seeded on both sides of PLCL scaffolds. The cells were seeded into the scaffold in a small amount of medium in order to be certain of getting the cell on the scaffold. The cells were allowed to adhere for 2.5 h, after which 500 μl of EpiLife medium or BM was added to vaginal EC or SC wells, respectively. The medium was changed three times per week and the cell-seeded scaffolds were cultured at 37°C in a humidified atmosphere until analyses. The analyses for vaginal EC and SC cultures were performed after 1, 7 and 14 days of cell culturing.

For co-culture wells, 40 000 SCs were implanted on scPLCL and pre-cultured for 5 days in BM in order to increase the SC amount. Thereafter, 40 000 ECs were implanted on the other side of the scaffold and the co-cultures were maintained in EpiLife medium at 37°C in a humidified atmosphere until analyses and the medium was changed three times per week. The analyses for co-cultured cells were performed at 1, 7 and 14 day time points, which are following the culturing time of vaginal ECs. The SCs were allowed to proliferate on serum containing medium for 5 days before implanting the vaginal ECs; thus, the corresponding cell culture times for SCs both in separate and in co-cultures were 6, 12 or 19 days.

### 2.6. Scanning electron microscopy imaging

The scanning electron microscopy (SEM) was used to evaluate the attachment and morphology of the ECs and SCs in co-cultures at 1, 7 and 14 day time points. Additionally, the SCs were evaluated after 1-day pre-culturing. Briefly, the cells were washed with Dulbecco's phosphate-buffered saline and fixed with 5% glutaraldehyde (Sigma-Aldrich) in 0.1 M phosphate buffer (pH 7.4, Sigma-Aldrich) at room temperature for 48 h. Thereafter, the samples were dehydrated through a sequence of increasing concentrations (30, 50, 70, 80, 90, 95 and 100%) of ethanol for 5 min. For drying, the samples were transferred into a solution of 1 : 2 hexamethyldisilazane (HMDS, Sigma-Aldrich) and 100% ethanol (Altia Oyj, Helsinki, Finland) for 20 min following an incubation in 2 : 1 HMDS and ethanol for 20 min. Thereafter, the samples were dried twice in 100% HMDS for 20 min. Finally, the samples were allowed to evaporate in a fume overnight, gold sputtered and examined with SEM (Zeiss ULTRAplus, Oberkochen, Germany).

### 2.7. Live/dead staining and cell proliferation

The viability of ECs, SCs and co-cultured cells was evaluated with qualitative live/dead staining at 1, 7 and 14 day time points, which was performed as previously represented by Sartoneva *et al.* [31] with minor modifications. The scPLCL without cells was used to exclude false-positive staining caused by material.

The DNA amount of both ECs and SCs cultured on scPLCL (*n* = 9) was determined using quantitative CyQUANT Cell Proliferation Assay kit (Invitrogen, Paisley, UK) at 1, 7 and 14 day time points as previously described by Vuornos *et al*. [25].

### 2.8. Immunostaining in co-cultures

The immunostaining was used to evaluate the expression of cytokeratins (AE1/AE3 pancytokeratin, 1 : 250, Cytokeratin Pan Ab, Thermo Scientific) and actin cytoskeleton organization (phalloidin-tetramethylrhodamine B isothiocyanate, 1 : 500, Sigma-Aldrich) in co-cultures of ECs and SCs at 7 and 14 day time point. Briefly, the samples were fixed with 4% paraformaldehyde (Sigma-Aldrich) and incubated overnight in pancytokeratin primary antibody dilutions. The next day, the samples were incubated in a mixture of secondary antibody (1 : 800 Alexa-488 donkey, green fluorescence) and phalloidin. Finally, the cell nuclei were stained with DAPI (1 : 2000, blue fluorescence, Sigma-Aldrich), and the cells were imaged with a fluorescence microscope (Olympus).

### 2.9. Real-time quantitative polymerase chain reaction

The relative expression of cytokeratin (CK) 7, CK8, CK19, uroplakin (UP) Ia, UPIb and UPIII genes was studied using real-time reverse transcription–polymerase chain reaction (qRT–PCR). For the qRT–PCR analysis, the ECs and SCs were cultured on scPLCL until 14 day time point. The ECs and SCs cultured on polystyrene (PS) served as a control. Briefly, total RNA was isolated with Nucleospin kit reagent (Macherey-Nagel GmbH & Co. KG, Düren, Germany). Thereafter, the mRNA was reverse transcribed to cDNA with the high-capacity cDNA Reverse Transcriptase Kit (Thermo Fisher Scientific). The expression of genes, CK7, CK8, CK19, UPIa, UPIb and UPIII was analysed and the expression data were normalized to the expression of housekeeping gene RPLP0 (large ribosomal protein P0). The primer sequences and the accession numbers are presented in table 1 (OligomerOy, Helsinki, Finland). The qRT–PCR mixture contained cDNA, forward and reverse primers, and SYBR Green PCR Master Mix (Applied Biosystems, CA, USA). The AbiPrism 7000 Sequence Detection System (Applied Biosystems) was used to conduct the reactions. The initial enzyme activation was performed at 95°C for 10 min, followed by 45 cycles at 95°C for 15 s and 60°C for 60 s. The relative expression was calculated using a previously described mathematical model [32].

**Table 1.** qRT–PCR primer sequences used in this study.

name		5′-sequence-3′	product size (bp)	accession number
CK7	forward	CATCGAGATCGCCACCTACC	80	NM_005556.3
	reverse	TATTCACGGCTCCCACTCCA		
CK8	forward	CCATGCCTCCAGCTACAAAAC	68	M34225.1
	reverse	AGCTGAGGTTTTATTTTGGACC		
CK19	forward	ACTACACGACCATCCAGGAC	80	NM_002276.4
	reverse	GTCGATCTGCAGGACAATCC		
UPla	forward	GGGATCTCCAGTTGGTGG	80	NM_007000.3
	reverse	TCTCAGCAAACAGGGACAGG		
UPlb	forward	AGTCACCAAAACCTGGGACAG	64	NM_006952.3
	reverse	TGATGGACCATTTACGCCACA		
UPIII	forward	TCAGTGCAAGACAGCACCAA	65	AB010637.1
	reverse	GTCCTCCCACCCTCTGTTTG		
RPLP0	forward	AATCTCCAGGGGCACCATT	70	NM_001002
	reverse	CGCTGGCTCCCACTTTGT		

### 2.10. Statistical analyses

Statistical analysis was performed with SPSS v. 23 (IBM SPSS Statistics for Windows, NY, USA). The cell proliferation measurement CyQUANT was repeated with three different cell lines using three parallel cell samples for each cell line (*n* = 9). The data inspection showed that the CyQUANT data were non-normally distributed; therefore, the differences between culturing periods were analysed using the non-parametric Kruskal–Wallis test. The qRT–PCR was done with three different cell lines using two or three parallel samples (*n* = 6). The non-parametric Mann–Whitney *U*-test, which analyses the differences between non-normally distributed data samples, was used to analyse the qRT–PCR data. Data from CyQuant and qRT-PCR were reported using median and quartiles; *p* < 0.05 was considered significant.

## 3. Results

### 3.1. Scaffold characterization

**Figure 1.**
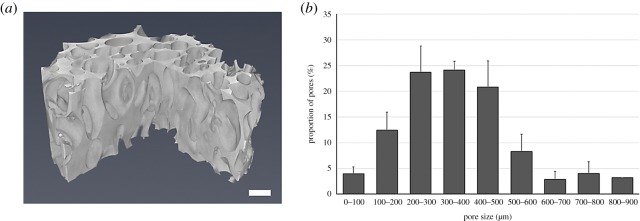
(*a*) The X-ray microtomography imaging illustrates the structure of porous PLCL scaffolds (scale bar, 500 µm). The column chart (*b*) is illustrating the pore size distribution of porous PLCL scaffolds. The average porosity is 65 ± 4% and the mean pore size is 350 ± 150 µm (*n* = 3).

The structure of three-dimensional scaffold is illustrated in figure 1*a*. The average porosity and average pore size of the three-dimensionally imaged samples were 65 ± 4% and 350 ± 150 µm, respectively (figure 1*b*). Interconnectivity of scaffold pores was 98% for a ball with 100 µm in diameter indicating that the 100 µm ball is able to reach 98% of the scaffold's total pore space from outside of the scaffold.

**Figure 2.**
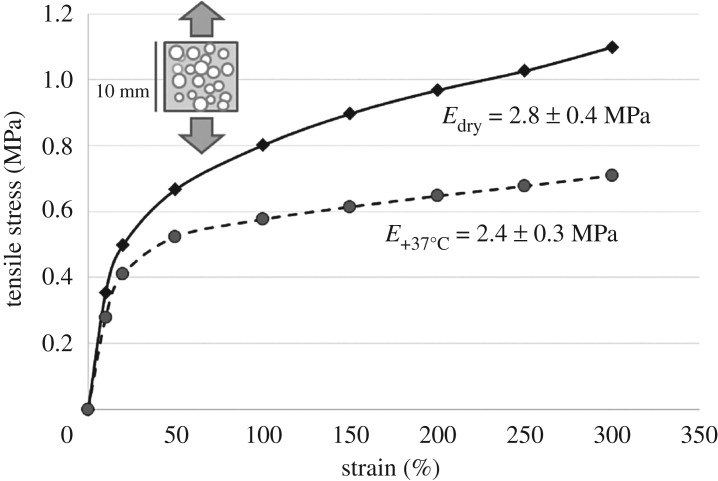
The stress–strain curves and elastic modulus of the tested dry (black line) and wet (dashed line) samples (*n* = 6).

The initial melting enthalpy (zero weeks) was 5.6 ± 0.9 J g^−1^ and after four-week incubation at 37°C, the melting enthalpy was 15.3 ± 0.8 J g^−1^. The elastic modulus for the dry samples in the ambient environment was 2.8 ± 0.4 MPa. After 2 days of incubation in phosphate buffer at 37°C, the elastic modulus was slightly decreased, being 2.4 ± 0.3 MPa. However, as presented in figure 2, the stress increase as function of strain was linear up to 20%, after which the slopes of the stress/strain curves changed to follow an environment-dependent new trend up to 300% strain. The materials did not break during the tensile test, but elastically returned to their original dimensions when the straining load was released. During the tensile test, the materials responded to even small dimensional changes in both ambient and in 37°C aqueous environment.

### 3.2. Flow-cytometric surface marker expression analysis

Based on the flow cytometric analysis, the populations of vaginal ECs and SCs were relative homogeneous between the donors. The vaginal ECs strongly (greater than 60%) expressed the cell surface molecules CD44, CD73, CD166 and CD326 (table 2). The moderate expression (20–60%) was detected on surface markers CD90 and CD105. The ECs illustrated low (2–20%) expression of CD133.

**Table 2.** Surface marker expression of vaginal stromal and epithelial cells after cell isolation. The expression level of greater than 60% was considered as strong, 20–60% as moderate and 2–20% as low expression.

cell type	donor	CD44	CD73	CD90	CD105	CD133	CD166	CD326
stromal cells	donor 1	100	100	99.9	99.9	1	98.8	7.2
	donor 2	100	100	99.8	99.9	1	96.9	13.3
	donor 3	100	100	99.6	99.9	1	97.3	12
epithelial cells	donor 1	99.4	100	18.6	69.8	0.8	99.9	95.2
	donor 2	95.9	99.9	21.5	52.1	3.2	99.7	99.3
	donor 3	94.7	100	61.4	44.2	2.9	99.7	87.5

The vaginal SCs strongly expressed the cell surface molecules CD44, CD73, CD90 and CD105. Additionally, strong expression of SCs was detected with CD166 and low expression of marker CD326 was detected with SCs. Further, the cells lacked (less than 2%) the expression of CD133.

### 3.3. Scanning electron microscopy

**Figure 3.**
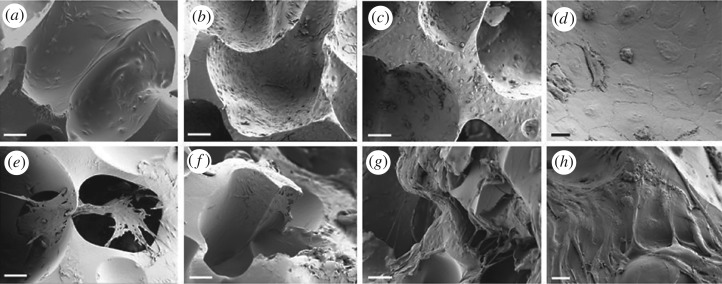
SEM images of the ECs and SCs in co-cultures on scPLCL at 1, 7 and 14 day time points. The SCs were pre-cultured in BM 5 days before seeding the ECs; therefore, the corresponding culturing times for SCs are 6, 12 and 19 days. (*a–c*) ECs at 1, 7 and 14 day time points, respectively (scale bar, 100 µm). (*d*) ECs at day 14, scale bar, 20 µm. (*e*–*g*) Fibroblasts at 1, 7 and 14 day time points, respectively (scale bar, 100 µm). (*h*) Fibroblasts at 14 day time point (scale bar, 20 µm).

SEM was used to examine the attachment and morphology of ECs and SCs in co-cultures on scPLCL (figure 3). After 1 day of cell seeding, the SCs were attached on scaffold surface (data not shown). The SCs were pre-cultured on scaffolds for 5 days before seeding the ECs. At 1 day time point, the vaginal SCs were cultured for 6 days and the vaginal ECs for 1 day on the scPLCL. The SCs were mostly adhered on the surface of the scaffolds; however, these cells also seemed to form cell cluster and cover the pores of the scPLCL. Further, the vaginal ECs were adhered on the surface of scPLCL. At 7 day time point, the ECs were spread homogeneously on the surface of the scPLCL, especially favouring the pores of the scaffold. At 14 days, the ECs had formed several layers (figure 3*c*) and were distributed evenly on the scaffold. At 7 and 14 day time points, the SCs favoured cell clusters and to form bridges between adjacent cells.

### 3.4. Cell viability and proliferation

**Figure 4.**
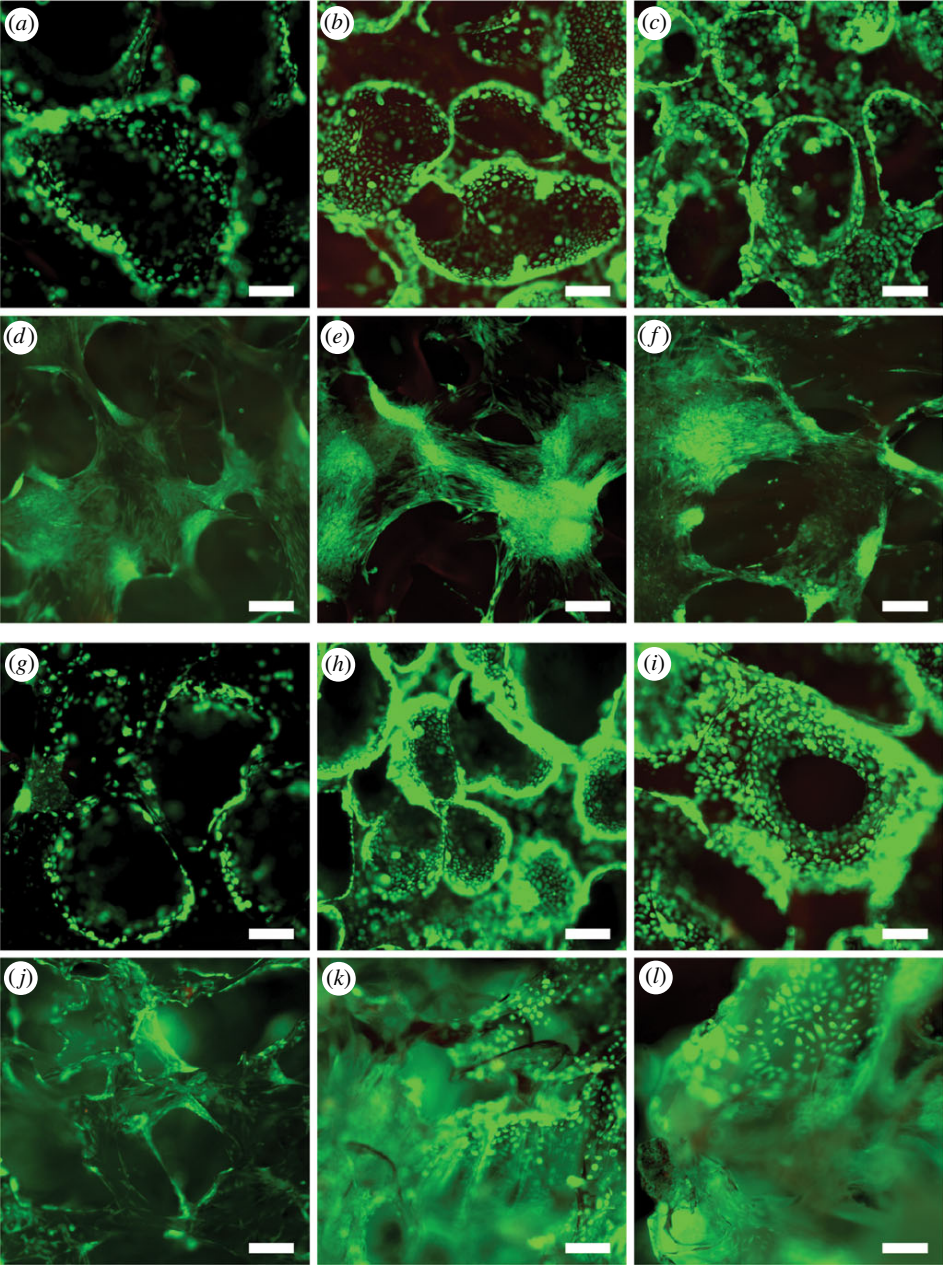
Live/dead images of ECs (*a–c*) and SCs (*d–f*) in separate cultures and of ECs (*g–i*) and SCs (*j–l*) in co-cultures at 1, 7 and 14 day time points, respectively. The majority of cells were viable (green fluorescence) and hardly any dead cells (red fluorescence) were detected on scPLCL both separate and co-cultures. Scale bar, 200 µm.

The viability of vaginal ECs and SCs and co-cultures was evaluated at 1, 7 and 14 days. Both vaginal ECs and SCs remained viable on scPLCL during the assessment period and the amount of dead cells was negligible. Further, the minor amount of dead cells after 1 day also indicates that the cells did not suffer from the 2.5 h adhesion period. Furthermore, the ECs and SCs were viable in co-cultures and no increase in the amount of dead cells was detected after 14 day time point (figure 4).

**Figure 5.**
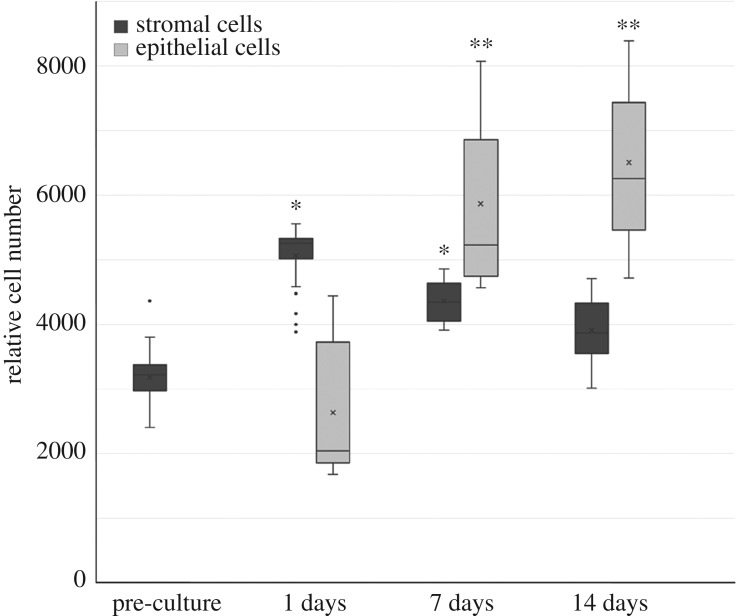
The proliferation of ECs and SCs in separate cultures on scPLCL was evaluated with quantitative CyQUANT cell proliferation assay. The amount of ECs increased during the assessment period (1 versus 7 days and 1 versus 14 days, *p* < 0.05, marked with **). The SCs were pre-cultured 5 days before epithelial cell seeding; therefore, the corresponding culturing times for SCs are 6, 12 and 19 days. The amount of SCs was significantly increased in 1 and 7 day time points when compared with pre-culture (1 day after sell seeding; *p* < 0.05, marked with *).

The cell proliferation in separate cultures was quantified based on DNA amount (figure 5). The proliferation assay indicated that the amount of vaginal ECs increased during the 14-day assessment period and the cell amount was significantly increased after 7 and 14 days when compared with 1 day. However, the amount of vaginal SCs reached the maximum at 1 day time point, after 6 days of cell culturing. The amount of SCs was significantly higher after 6 and 12 days of cell culturing compared with 1 day after cell seeding.

### 3.5. Immunostaining in co-cultures

**Figure 6.**
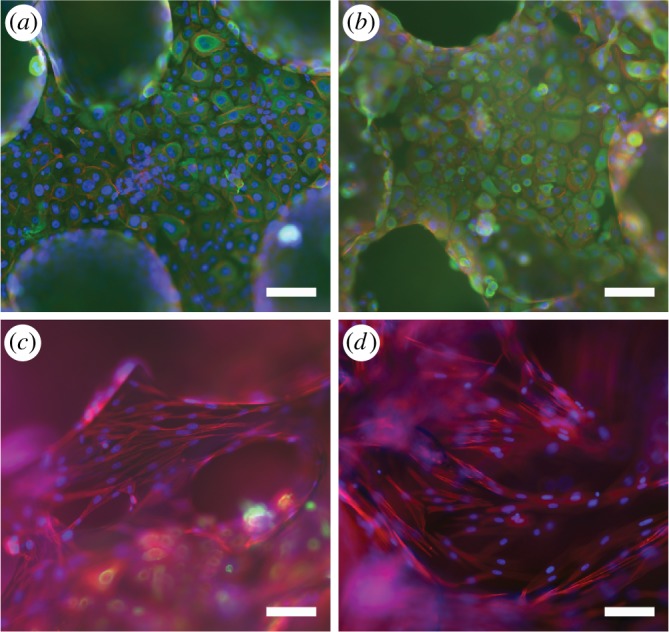
Immunostaining images of co-cultured ECs (*a,b*) and SCs (*c,d*) on scPLCL. The ECs (*a,b*) strongly express the cytokeratins (pancytokeratin, AE1/AE3) at both 7 and 14 day time points. Further, the actin cytoskeleton organization of vaginal ECs or SCs did not change during the assessment period.

The expression of cytokeratins (pancytokeratin AE1/AE3) and organization of actin filaments (phalloidin) were assessed in vaginal ECs and SCs co-cultures at 7 and 14 day time points (figure 6). The vaginal ECs expressed cytokeratins strongly in both time points and there was no remarkable alteration in the expression intensity. Further, the cytokeratin expression was absent in vaginal SCs. In SCs, the actin filaments were aligned in parallel, whereas, in the ECs, the actin seemed to accumulate on the edge of the cell cytoskeleton.

### 3.6. Real-time quantitative polymerase chain reaction

**Figure 7.**
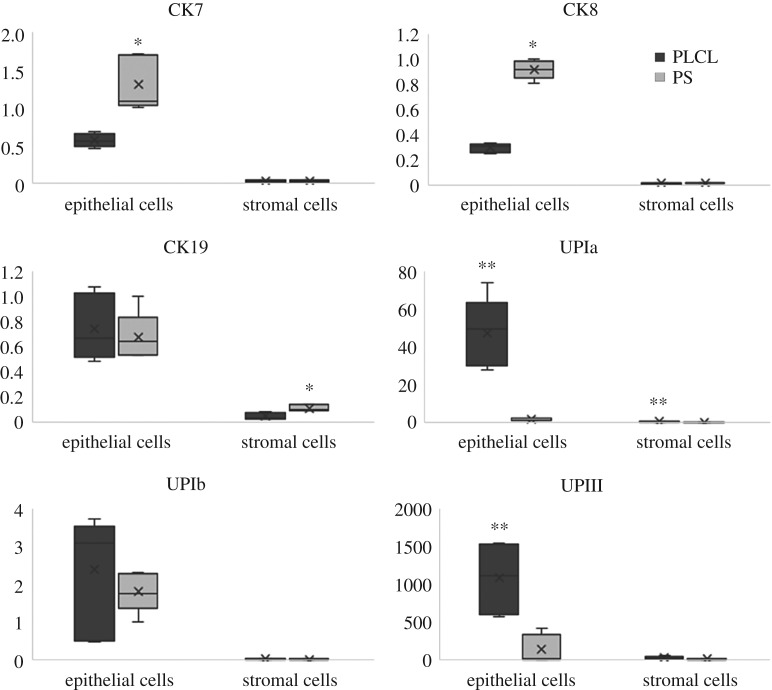
The expression of markers CK7, CK8, CK19, UPIa, UPIb and UPIII were evaluated after 14-day assessment period with ECs or SCs cultured on scPLCL. The cell culture plastic (PS) served as a control. ECs on PS expressed the markers CK7 and CK8 significantly more compared with scPLCL (*p* < 0.05, marked with *), whereas the marker UPIII was superiorly expressed on epithelial cells cultured on PLCL (*p* < 0.05, marked with **). Both ECs and SCs expressed the marker UPIa superiorly on scPLCL compared with PS (*p* < 0.05, marked with **).

The expression of epithelial markers was studied at 14 day time point in vaginal ECs cultured on scPLCL and the PS, which served as a control material (figure 7). The vaginal ECs expressed all the cytokeratin markers, CK7, CK8 and CK19. The expression of CK7 and CK8 was significantly lower on PLCL scaffold compared with PS, whereas the CK19 expression was similar on both materials. Interestingly, the scPLCL supported the gene expression of UPIa and UPIII significantly more compared with PS in vaginal ECs. In addition, we evaluated the expression of the same epithelial markers in vaginal SCs cultured on scPLCL or PS. The SCs lack or indicated only low expression of cytokeratin and uroplakin genes on both scPLCL and PS cell culture wells.

## 4. Discussion

The surgical reconstruction of vaginal tissue is highly challenging and susceptible to complications. Various non-vaginal tissues such as skin grafts, intestinal tissue, vulvar skin flaps or inverted penile skin have been used to reconstruct a neovagina. However, it has been reported that neovagina contains higher risk for malignancy and the type of cancer seems to be related to the transplanted tissue [33,34]. The rising numbers of transgender reconstruction, the advancing cancer detection and treatment, and the modern expectations for the quality of life will bring more patients in need for safe and efficient treatment methods for vaginal deformity [35,36]. Owing to these challenges, it is highly important to develop new treatment techniques.

Previously, both natural and synthetic materials have been tested for vaginal tissue engineering [37–39]. In general, the natural biomaterials are highly biocompatible and contain natural cell adhesion molecules. However, natural biomaterials are usually xenogenic, which could elicit immunological reactions and contain a minor risk of viral infection transmission. In comparison, the synthetic materials are less bioactive but large-scale manufacturing of even quality products is fairly easy and the mechanical properties are more easily tailored. Previously, DeFilippo *et al*. studied vaginal epithelial and smooth muscle cells cultured on poly-lactide-co-glycolide-coated polyglycolic acid scaffold in a rabbit model. They compared the tissue-engineered scaffold to unseeded scaffold for vaginal reconstruction in a rabbit model. After six months, the tissue-engineered vagina revealed normal histology and maintained the vaginal calibre, whereas, on unseeded scaffold group, the strictures or graft collapse occurred one month after surgery [37]. In addition, Raya-Rivera *et al*. have treated four patients suffering from vaginal aplasia using small intestinal submucosal graft seeded with patient's own epithelial and SCs acquired from vulvar biopsy. The histology of neovagina remained normal in yearly biopsies over the 5–8-year follow-up period; also, the variables in the sexual function index questionnaire remained in normal range with all patients and no long-term post-operative complications were reported [39]. Despite encouraging results, there are only a few studies in the field of vaginal tissue engineering.

For this study, we isolated normal vaginal ECs and SCs from vaginal biopsy, after which the cells were characterized in order to control the success of our isolation protocol. At best, to our knowledge, vaginal ECs or SCs have not been previously characterized with flow cytometry. Thus, this study provides novel findings on the CD surface marker expression pattern of these cells. The vaginal ECs strongly expressed the extracellular matrix adhesion molecule CD44, surface molecule CD73 and epithelial markers CD166 and CD326. Previously, we have detected that these markers are strongly expressed in urothelial cells; therefore, we assumed that these markers would also be expressed in vaginal EC [20,31,40]. The vaginal SCs strongly expressed the markers CD44, CD73, CD90 and CD105, illustrating the mesenchymal origin of the SCs [41,42]. Additionally, the strong expression of marker CD166, which is known to be expressed also in fibroblast cells, was also detected in vaginal SCs [43]. The differences in CD marker expression profile also showed that the isolated vaginal EC and SC populations were different, indicating the success of our cell isolation protocol.

According to our knowledge, neither PLCL nor scCO_2_ foaming as a scaffold manufacturing method has been previously studied for vaginal tissue engineering. The scaffold for vaginal tissue engineering should be elastic, easy to form and suture and mimic the properties of natural extracellular matrix as closely as possible. Lei *et al*. [44] have compared the mechanical properties of normal and prolapsed vaginal tissue and according to their result, the elastic modulus of normal vaginal tissue in pre-menopausal women is 6.65 ± 1.48 MPa. According to our results, the elastic modulus of scPLCL after 2 days *in vitro* was 2.4 ± 0.3 MPa, which can be considered sufficient for vaginal tissue engineering application because too stiff scaffold may lead to biomaterial erosion [37,44,45]. Our tensile test demonstrated the high elasticity of scPLCL, which is important property for vaginal as well as other soft tissue engineering applications. Further, the results with scPLCL are comparable with previous results for PLCL [20,24,46,47]. The plasticizing effect of water in combination of hydrolytic degradation increased the molecular movement in aqueous environment at 37°C and increased crystallinity of the scCO_2_-foamed scaffold [48]. The crystallinity after 4-week incubation was at the same level as previously reported [49], but due to porous structure, the mass loss and overall degradation profile may differ and thus has to be investigated in future studies.

We evaluated the viability and proliferation of vaginal ECs and SCs on scPLCL. We also studied how the vaginal ECs and SCs attach and retain their morphology and viability in co-cultures. The PLCL is known to be a biocompatible material [20,21,24], which is further demonstrated in this study, because both vaginal ECs and SCs maintained their viability and proliferated in separate cultures during the 14-day culturing period. The scPLCL seemed to support especially the proliferation of ECs and the cell amount increased during the whole 14-day assessment period. Interestingly, the SCs reached their proliferation maximum after 6 days of cell culturing and the amount of cells slightly decreased after that. Moreover, the cells remained viable in vaginal EC and SC co-cultures and no increase in dead cell amount was detected. The SEM imaging showed that the ECs attached and spread evenly on the scPLCL and especially the ECs seemed to favour the pores. In addition, after 14 days, the vaginal ECs seemed to form layers. At 7 and 14 day time points, the vaginal SCs seemed to form clusters and were not so evenly distributed on the surface of the scaffold as ECs, at least in co-culture conditions, suggesting that PLCL supports the vaginal ECs over the SCs. However, this might be due to the used medium because epithelium medium, EpiLife, was used in co-cultures after EC seeding.

We used qRT–PCR to study the gene expression of CK7, CK8, CK19, UPIa, UPIb and UPII in vaginal ECs and SCs after 14 days of cell culture on the scPLCL, cell culture plastic (PS) serving as a control. The CKs are cluster of intermediate filament proteins, which are widely expressed in EC. Based on the results of this study, CK7, CK8 and CK19 are also expressed in vaginal ECs, as expected [50–52]. Interestingly, the expression of CK7 and CK8 was lower on scPLCL compared with PS, whereas the CK19 expression was similar in both materials. The UPs are a group of transmembrane proteins, which are expressed in urothelial cells and are mainly present in the superficial urothelial cells; however, UPs may also have a role in the urogenital tract and vaginal development [53–55]. Therefore, we wanted to study if the vaginal ECs express the UP genes. Interestingly, we detected that the vaginal ECs expressed UPIa and UPIII markers significantly more on scPLCL compared with PS, whereas the SCs seemed to express marker UPIa significantly more on scPLCL compared with PS.

In addition to gene expression, we also studied the maintenance of CK expression and actin cytoskeleton arrangement by phalloidin staining on scPLCL at 7 and 14 day time points in vaginal EC and SC co-cultures. The actin filaments in SCs were parallel aligned according to the cell cytoskeleton and in ECs, the actin filament staining was detected on the edge of the ECs. However, no remarkable changes were detected in the actin organization between the time points. The pancytokeratin staining showed that ECs expressed cytokeratins equally in both time points indicating that the cells remain their epithelial phenotype during the co-culture; further, the SCs lacked the expression of cytokeratins marker. Thus, both vaginal ECs and SCs seemed to maintain their phenotype during the assessment period.

## 5. Conclusion

Both vaginal ECs and SCs retained their viability, phenotype and morphology during the assessment period both in separate and in co-culture conditions in scPLCL scaffolds. Hence, this study demonstrates that scPLCL is a potential scaffold material for vaginal tissue engineering further illustrating the good biocompatibility of PLCL. The Epilife medium, which was used in the co-cultures of ECs and SCs, was not optimal for SCs. Therefore, it is highly important to further develop cell culture conditions and medium compositions that are more favourable for EC and SC co-cultures.

## Supplementary Material

Medium composition testing with live/dead staining

## Data accessibility

Data supporting this research are uploaded as electronical supplementary material or available from Dryad: http://dx.doi.org/10.5061/dryad.2bg877b [56].
