# Supplementary material for: Porous poly-l-lactide-co-ɛ-caprolactone scaffold: a novel biomaterial for vaginal tissue engineering
Source: R Soc Open Sci. 2018 Aug 15;5(8):180811. doi: 10.1098/rsos.180811 (PMC6124079; doi:10.1098/rsos.180811)
Supplement: Medium composition testing with live/dead staining [file rsos180811supp1.doc]

**Electronical supplementary material**

**Porous poly-l-lactide-co-ε-caprolactone scaffold: a novel biomaterial for vaginal tissue engineering**

Reetta Sartoneva^1,2*^, Kirsi Kuismanen^2,3,4*^, Miia Juntunen^1,2^, Sanna Karjalainen^5^, Markus Hannula^6^, Laura Kyllönen^1,2^, Jari Hyttinen^6^, Heini Huhtala^7^, Kaarlo Paakinaho^5^, Susanna Miettinen^1,2^

^1^Adult Stem Cell Research Group, BioMediTech, Faculty of Medicine and Life Sciences, University of Tampere, Tampere, Finland

^2^Science Centre, Tampere University Hospital, Tampere, Finland

^3^Department of Obstetrics and Gynaecology, Tampere University Hospital, Tampere, Finland

^4^Faculty of Medicine and Life Sciences, University of Tampere, Tampere, Finland

^5^Biomaterials and Tissue Engineering Group, BioMediTech, Faculty of Biomedical Sciences and Engineering, Tampere University of Technology, Tampere, Finland

^6^Computational Biophysics and Imaging Group, BioMediTech, Faculty of Biomedical Sciences and Engineering, Tampere University of Technology, Tampere, Finland

^7^Faculty of Social Sciences, University of Tampere, Tampere, Finland

* Equal contribution

Corresponding author: Reetta Sartoneva, MD, PhD, MSc

University of Tampere, BioMediTech

Arvo Ylpönkatu 34, 4^th^ floor, 33520 Tampere, Finland

Email: reetta.sartoneva@fimnet.fi

Phone: +358 40 024 5371

**Figure S1**

**
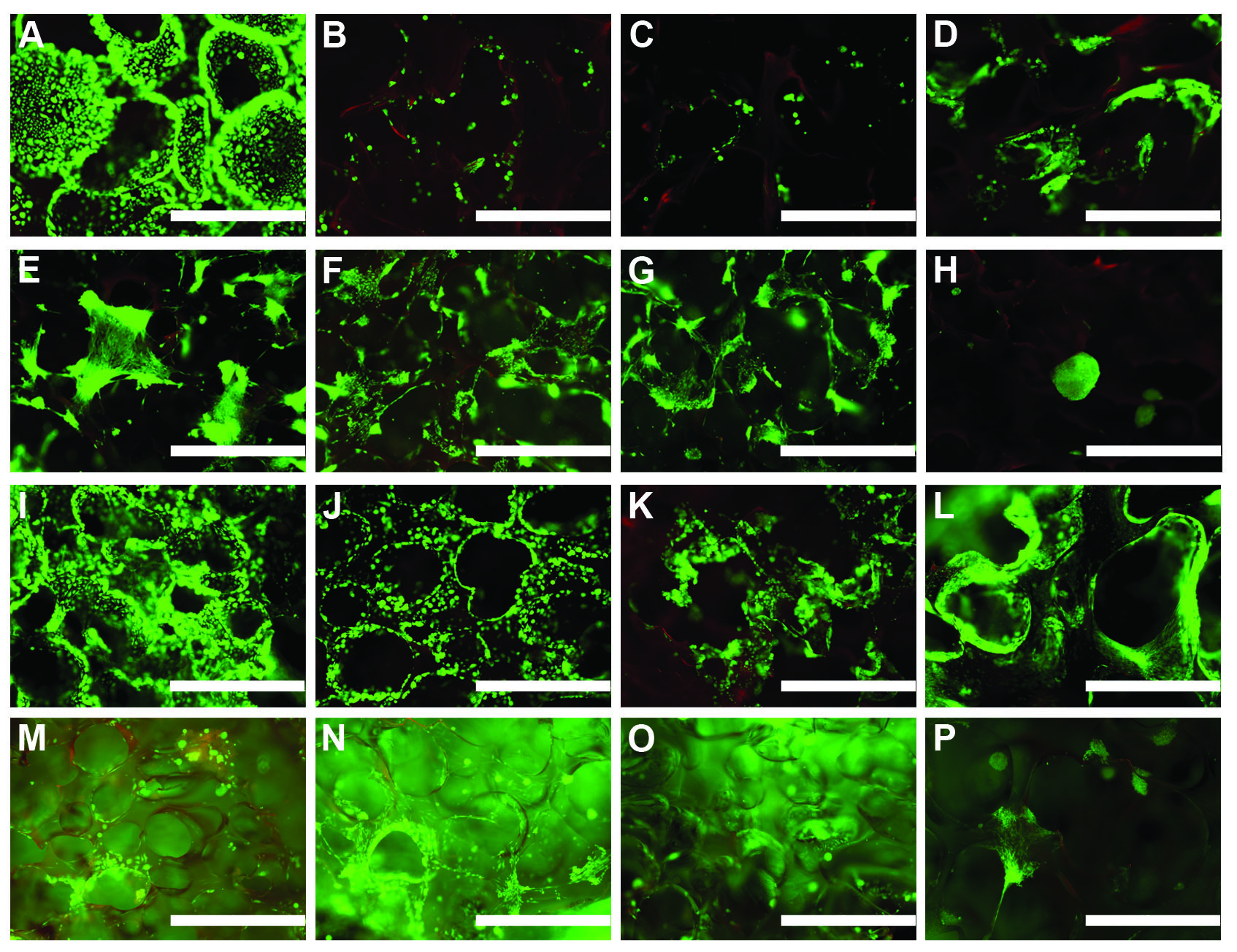
**

Figure S1: Medium testing live/dead images at day 7. The epithelial cells (A-D), stromal cells (E-H) and their co-cultures (I-P) were cultured in different medium compositions; EpiLife (A,I,M) or 5% HS in DMEM/F12 (E),  3 % HS in EpiLife (B,F,J,N), EpiLife  and  BM  1:1 (C,G,K,O) and CnT prime CC (D,H,L,P). Scale bar 1 mm.
